# Recommendations for increasing the use of HIV/AIDS resource allocation models

**DOI:** 10.1186/1471-2458-9-S1-S8

**Published:** 2009-11-18

**Authors:** Arielle Lasry, Anke Richter, Frithjof Lutscher

**Affiliations:** 1 Department of Mechanical and Industrial Engineering, University of Toronto, Toronto, Canada; 2 Defense Resources Management Institute, Naval Postgraduate School, Monterey, CA, USA; 3 Department of Mathematics and Statistics, University of Ottawa, Ottawa, Canada

## Abstract

**Background:**

Resource allocation models have not had a substantial impact on HIV/AIDS resource allocation decisions in spite of the important, additional insights they may provide. In this paper, we highlight six difficulties often encountered in attempts to implement such models in policy settings; these are: model complexity, data requirements, multiple stakeholders, funding issues, and political and ethical considerations. We then make recommendations as to how each of these difficulties may be overcome.

**Results:**

To ensure that models can inform the actual decision, modellers should understand the environment in which decision-makers operate, including full knowledge of the stakeholders' key issues and requirements. HIV/AIDS resource allocation model formulations should be contextualized and sensitive to societal concerns and decision-makers' realities. Modellers should provide the required education and training materials in order for decision-makers to be reasonably well versed in understanding the capabilities, power and limitations of the model.

**Conclusion:**

This paper addresses the issue of knowledge translation from the established resource allocation modelling expertise in the academic realm to that of policymaking.

## Background

Resource allocation models can provide valuable guidance to the rational allocation of funds. Improving the use of funds by targeting areas of greatest return may lead to better and/or greater health outcomes. Although there is growing pressure to use formal modelling techniques in healthcare [1], their impact on healthcare resource allocation has been rather limited [2-4]. In addition, there is evidence that actual spending decisions tend to deviate from what formal models might suggest [5]. The decision-making process for HIV/AIDS resource allocation is a combination of several components, including historical spending patterns, influential players such as advocacy group or donors, priority setting assessments and formal models. Thus, resource allocation models are but one component of a broader allocation process. We argue that healthcare modellers can benefit from a comprehensive understanding of all components of a resource allocation process in order to improve model acceptance among decision-makers and the contribution of models to the overall process.

This article explores several factors that tend to prevent the application of formal resource allocation models and provides recommendations as to how these barriers to implementation can be overcome.

## Overview of HIV/AIDS resource allocation models

Resource allocation is defined as the distribution of resources among programs, populations or regions that are competing for the same funds [6]. Several types of approaches have been employed in creating resource allocation models for HIV/AIDS interventions funds, and methodologies have evolved over time. Initial models employed basic methodology such as the allocation of funds proportional to the number of HIV and/or AIDS cases in target groups [7,8] and allocation choices determined by ranking cost-effectiveness league tables [5,9,10]. From this starting point, there have been continual refinements in modelling techniques. The first of these refinements focused on the underlying epidemic with complex dynamic models constructed to reflect the impact of resource allocation choices on the spread of disease [11]. More recent models implement simulation optimization techniques or non-linear programming to determine optimal allocation for epidemiological models of varying degrees of sophistication [12]. Another means of refinement involved direct modelling of current funding strategies within the resource allocation model [13,14]. Others have focussed on determining the optimal location of facilities for maximizing the accessibility of antiretrovirals [15-17].

The emphasis on techniques may reflect several aims within the research community. There is a requirement for greater accuracy in the epidemic projections used as a basis for resource allocation. Scientific accuracy can help withdraw HIV/AIDS resource allocation issues from the political arena and minimize subjectivity in the allocation process. Politics and value judgments tend to be at odds with rational comprehensive decision-making processes for allocating of resources [18-20]. However, highly complex, difficult to understand and use, formal models have led to difficulties in the actual implementation of these models in real-world decision-making.

## Discussion

In this section we discuss the main barriers to the adoption of HIV/AIDS resource allocation models and provide recommendations for overcoming these barriers.

### Model complexity

Barriers to the use of formal models in healthcare include decision-makers' lack of appreciation and understanding of models and their conclusions [21]. Decision-makers should understand the conceptual foundation of a model and its calculations to gain confidence in its results and implement its recommendations, especially when these seem counterintuitive.

Relatively few resource allocation publications appear in medical or public policy journals compared to the body of resource allocation literature published in quantitative journals. This difference underscores the lack of knowledge transfer from healthcare modelling to decision-making. Published examples documenting the lack of model usage are difficult to find. We identified only one commentary by Kaplan on an HIV resource allocation model designed for community planning groups [22]. The model was not implemented because the intended users did not comprehend or accept it or its results [22].

Some suggest that modellers tend to focus on the sophistication of the mathematical formulation of models for the control of sexually transmitted diseases rather than on practical relevance [23]. Given the common lack of mathematical knowledge among decision-makers, sophisticated models tend to widen the gap between modellers and the decision-makers originally intended to use the models [23]. Stover suggests that mathematical models of HIV/AIDS have had little influence on policies in developing countries because decision-makers believe that models are incomprehensible and do not yield realistic recommendations [24]. This latter point is exacerbated by the fact that most models do not consider the potential synergism or antagonism resulting from the interaction of several types of interventions typically conducted simultaneously within a single community. While models could consider these interactions, there is a lack of reliable data to support them. Finding means to address these issues to the satisfaction of the policymaker and the modeller is important for model usage.

#### Recommendations

Resource allocation models should be considered a process to be undertaken by all parties involved, rather than a product to be delivered by the modeller. Modellers need to fully understand the requirements of their potential end users as well as the decision-making environment in which these users function. Model development should proceed from simple to increasingly complex, with consultations between modellers and end users to determine the appropriate level of complexity of each aspect of the model. Modellers should arrange for education and training materials in order for users to be reasonably well versed in the features, power and limitations of the model. Documentation should be in language that is clear to the intended users; this might require different documentation for different users of the same model. Graphical representations tend to avoid the need for extensive mathematical notation. End users should provide the modeller with necessary information for model development in a timely manner and be open to question their own views and assumptions of the process to be modelled. When there are multiple groups of potential users, discussion on the model formulation and structuring is pivotal to developing consensus. To expedite and ease these discussions, each group of intended users should designate one individual as a "translator" or "spokesperson" between the modeller and the group. Also, when there is a team of modellers, a spokesperson should be designated and selected based the ability to communicate complex mathematical methods in a logical and accessible manner. Frequent discussions with the intended users will ensure that the model is relevant and comprehensible.

### Data requirements

Context-specific data requirements for models can be onerous. The Institute of Medicine cites a lack of resource allocation frameworks that rely on existing data [25]. Many HIV/AIDS resource allocation models require information that is not available or collected by governmental or research agencies, such as behavioural data of risk groups within a community. In the absence of data, assumptions must be made. However, decision-makers are wary of models lacking requisite supporting data since this lack could lead to unfortunate decisions. For example, should behavioural patterns observed in the general population be applied to a high risk group due to a lack of available data, then interventions in that group may not seem cost-effective even if, in reality, they are [26]. Decision-makers' uncertainty about data estimates and assumptions can lead to a wholesale rejection of decision-support models.

In addition to information about epidemic propagation, HIV resource allocation models often rely on intervention cost and effectiveness data which is difficult to obtain. The CDC published the Compendium of HIV Prevention Interventions which contains a list of interventions deemed effective but it does not provide any cost estimates [27]. Some cost data are available in the published literature, but they tend to be specific to the community in which the intervention was implemented and cannot easily be transposed to a different context. Intervention program costs and outcomes are affected by local considerations such as transportation availability, amount of public healthcare delivery, community mobilization and socio-economic status. Holtgrave states that when "cost-effectiveness information does not exist for a particular population, the real potential exists for that population to be put at a disadvantage in the resource allocation process." (p.184)[28]. In 1993, Birch and Gafni stated that some assumptions used in cost-effectiveness analysis are "worlds apart from the problem context facing decision-makers in the healthcare field" [29]; in a subsequent paper in 2006 they reiterate that cost-effectiveness analyses are often misused in resource allocation [30].

#### Recommendations

Modellers should understand data availability *a priori *and develop tools that function with the available data. For example, if program cost or outcome data are not available, then resource allocation approaches such as prevalence- or incidence-based equity models that do not rely on these data should be considered. If critical data gaps are identified, decision-makers may be called upon to collect some of the necessary data either through secondary sources or by conducting their own surveys. However, this requires careful balancing of the improvement in resource allocation that may be attainable with better data and the amount of time and resources that must be expended to collect this data. Modellers and policymakers should update model inputs and regenerate results as more accurate and recent data become available.

Sensitivity analyses are arguably the most powerful and most underused tools that a modeller has to address these issues. Sensitivity analysis explores the extent to which changes in input data affect the model outputs by systematically varying data values and model assumptions. Their use is critical since they can determine whether it is necessary and effective to gather more information. If the recommendations resulting from the model do not change over the expected range of values, then decision-makers can feel more certain of the resource allocations recommended by the models. Sensitivity analyses should not be presented as an afterthought in the model development process, but rather explained and touted as an integral part of the decision-making process. Repeated use of sensitivity analyses to inform decisions about increasing model complexity can save time and money in the model-development process.

### Multiple stakeholders

In any decision process, multiple stakeholders can affect a model's development and its acceptability. Resource allocation models may encounter barriers to use when they optimize the expected outcome from the perspective of one decision-making body [31]. Different stakeholders take part in the decision-making process for HIV/AIDS resource allocation, each with their own objectives, constraints and realities; they include donors, lobby groups, community-based organizations and national, state or local governments. Eden and Ackerman define a matrix of the amount of power and interest different stakeholders have on the decision problem [32]. There are the "Subjects" - those with high interest in the decision but little power to affect the decision, the "Players" - those with both high interest and high power to affect the decision, and the "Context Setters" - those with high power to affect the decision but with relatively little direct interest in the decision itself. Players may regard models with suspicion when they are not consulted in the model building process. Subjects can band together to gain political clout as a Player, as seen in the rise of HIV/AIDS advocacy groups. They may reject results that appear to be putting their interests at a disadvantage. Players must interact with Context Setters who frequently have to make the final decisions. Belton and Stewart remark upon similar results seen in other arenas [33].

There is also debate on how health benefits and risks are assessed within the resource allocation models - by the affected individuals, by representatives, by experts. Quality of life assessments vary widely between patients, physicians and the general public [34-38]. Therefore, the point of view of the assessor of the benefits can dramatically affect cost-effectiveness analyses. In addition, it is not clear whether or not all patients should be viewed as a homogenous group [34]. Treatment costs of HIV/AIDS and quality-adjusted life years saved will vary by socio-economic characteristics; there is debate as to whether or not these should be racially specific or reflective of the general population [28,39]. Michaelis notes that the decision to commit resources to AIDS started very slowly "because the primary victims were socially marginalized or economically disadvantaged" (p. 400) [40].

#### Recommendation

It is important to understand stakeholders' needs and influences, and engage them early in the modelling process to increase their willingness to cooperate [41]. The cooperation of stakeholders is especially important since maximizing the health benefits of society are frequently not the same as maximizing the health benefits of the individual [35,42,43]. Exploring and potentially incorporating some of the alternate means of achieving distributive justice is critical for model acceptability, since it is perceived as an important value representing fairness and equity [35,44,45].

When multiple players influence decision-making, resource allocation models are meant to help structure the problem and promote conflict resolution in a constructive way [31]. One of the powers of mathematical models is that all assumptions have to be made explicit before the model can be implemented and analyzed. The modeller has a key role of pointing out potentially differing assumptions and/or goals of all groups involved.

In addition, for models to be accepted by multiple stakeholders, the variables that serve as input to the model - for example, treatment access, life years saved, quality adjustments and costs - need to be discussed, documented and made transparent for review. Again, sensitivity analysis is key tool to exploring how different values or relative weights of these inputs influence the outcome of the model.

### Political considerations

While modellers may distance themselves from political considerations so as to avoid bias in their research, resource allocation decisions are inevitably influenced by the politics in the budget appropriations environment. HIV/AIDS is particularly prone to political debate in part due to the sexual nature of the disease, involvement of injection drug use, and the high cost of care and treatment.

In South Africa, the response to the HIV/AIDS has been riddled with political hurdles. President Thabo Mbeki's dissident views on HIV/AIDS have been widely reported [46-49]. Parts of the political leadership in South Africa have cast doubt on the causal link between HIV and AIDS, and the efficacy and affordability of antiretroviral treatment [50]. The Treatment Action Campaign (TAC), an advocacy group, was launched to advance the provision antiretroviral treatment for people with AIDS. In August 2003, motivated by the TAC, the government consented to a plan that aimed to have 53,000 people on treatment by March 2004 [51]. In June 2005, while below this target, health minister, Dr. Tshabalala-Msimang said she refused to be "pressured" into increasing the antiretroviral roll-out to meet a target set by a group of international bodies without consulting South Africa on its feasibility. She also reiterated her praise of garlic and lemon: "not only do they give you a beautiful face and skin but they also protect you from disease" [52]. Leadership stance, political power, advocacy groups and the judicial system have largely influenced South Africa's allocation of HIV/AIDS funds.

Another example of the overriding impact of political considerations can be found in the US: an appropriations bill prohibiting the District of Columbia from using local tax dollars to operate needle exchange programs was in effect. The District has some of the highest HIV/AIDS rates in the country, with one third of its new HIV/AIDS cases annually attributed to injection drug use. Needle exchange programs are demonstrably effective in preventing HIV transmission, cost-effective [53], and do not generate unsafe disposal of needles [54]. When included in resource allocation models, needle exchange programs are some of the first that the models will recommend [55]. Washington DC was the only city prohibited from using local tax dollars to provide clean needles to IV drug users until the ban in was lifted in 2007 [56].

#### Recommendations

Resource allocation decisions should not be devoid of their socio-political environment [57]. It is difficult, even impossible to use the results of a rational model to guide allocation policies without consideration of the political context [47]. Though formal approaches to resource allocation models cannot alter this level of political power struggle, they can inform the process. Modellers must understand the political context and craft flexible models that allow for restrictions on portions of funds or other political constraints. Flexible models allow decision-makers to determine the best course of action within the politically constrained process as well as the best course if the political realities can be influenced and changed [20]. To illustrate the expected impact, the existing allocation of resources should be presented against the alternative allocations suggested by the model. By highlighting the most restrictive constraints, models may guide both the effort and the magnitude of the effort to lobby for change.

### Ethics and human rights

Social and ethical considerations include culture, religion, human rights, societal inter-relationships, and community advocacy; all of which are difficult to quantify. An example of the impact of these considerations can be seen in the debate on funding treatment versus prevention. Marseille et al. suggest that prevention be favoured in order to minimize the number of deaths from HIV/AIDS in sub-Saharan Africa [58]. Based on a meta-analysis of the cost-effectiveness of HIV/AIDS programs in Africa, Creese et al. also argue for prevention interventions [59]. Some disapproved of these economic studies on the basis that funding decisions should include the right to improved health for those already infected and that society has an ethical duty to treat those who are sick [60-62]. The latter point invokes the rule of rescue which states that denying treatment to an identified suffering person facing death is considered morally repugnant by most, while denying prevention to future sufferers who are a faceless statistical abstraction is more palatable [63].

The human rights versus public health dichotomy is well captured in the provocative example of Cuba where testing and contact tracing was compulsory, and from 1985-1994, diagnosed patients were isolated to a sanatorium for the HIV/AIDS infected [64,65]. In 2005, it is estimated that 4800 people in Cuba were living with HIV/AIDS representing a prevalence of less than 0.1%, while the average prevalence for the Caribbean is 1.6% making it the second most-affected region in the world after Africa [66]. Given that isolation is known to be an effective public health control measure, some may view the Cuban approach as a success. However, it has been strongly criticized from a human-rights perspective and many feel that such an approach would not be feasible in other countries [65].

Screening pregnant women for HIV initially generated much public discourse and demonstrates the clash between pragmatic public health and human-rights approaches. Mother-to-child transmission can be reduced dramatically when appropriate prevention measures are taken. Voluntary or anonymous testing of pregnant mothers puts newborns at risk because life-saving and cost-effective interventions cannot be offered should the mother refuse consent [67]. On the other hand, mandatory testing is seen as violating the mother's rights and civil liberties. In communities where HIV infection carries significant social stigma, an HIV diagnosis could lead to expulsion from society and the family unit. Here, the perceived benefits and rights of women to refuse testing (or disclosure of test results) are weighed against the risks to infants being exposed to the virus [68]. Again, models may have little ability to sway the discussion in this debate, regardless of their results or the technical competence with which they are constructed.

Another controversial issue revolves around whether targeting interventions to high risk groups, a classic public health approach, promotes fear and stigmatization, drives the epidemic 'underground' and in turn contributes to the problem [69]. The epidemiologic importance of high-frequency transmitter core groups was initially suggested by Hethcote and Yorke [70]. Cost-effectiveness of treatment and prevention programs can be high when programs are tightly targeted [71,72] to core groups such as commercial sex workers, injection drug users or men who have sex with men. When resource availability is low, funds should be targeted on changing the behaviour of those most likely to contract and spread the epidemic [73]. Most classical resource allocation models endorse this approach. However, sociology approaches do not measure success solely in terms of costs and effectiveness. Decosas argues that even public health terminology such as "risk groups" and "targeting" endorses stigmatization, which is difficult to undo, and that targeting core groups "once translated from the depoliticized discourse of epidemiology into the world of real people, takes on the meaning of controlling 'them' in order to protect 'us'" [74].

#### Recommendations

Resource allocation practices are subject to the influence of many social and ethical considerations that are not considered in purely rational models. While it may be impractical for models to consider these non-quantitative aspects, model applicability may be improved if the main drivers are determined and included in the model as options. Awareness and flexibility allows modellers to help the decision-maker assess the impact of several non-quantitative considerations and determine which are most important. Addressing social and ethical concerns in a proactive manner can enhance model acceptability, while ignoring them is a strong barrier to the adoption of models.

### Funding issues

Funding sources may present additional constraints on the ability of recipients to focus on their established priorities, on the use of certain interventions, on the ability to refocus funds from existing programs, and on the ability to disaggregate intervention-impact information.

There is a definite relationship between a country, locality or institution's degree of reliance on donor funding and the extent to which donors can influence resource allocation decisions. Some donors have fixed priorities and strict agendas. For example, in 2003 the President's Emergency Plan for AIDS Relief (PEPFAR) committed US$15 Billion to scaling up HIV/AIDS related services in countries that are most resource constrained. PEPFAR has strict monitoring and evaluation rules, and funds are generally not pooled with other donors - presumably so that the impact of the investment can be evaluated. Other donors, such as the Global Fund for AIDS, Tuberculosis and Malaria, are not as restrictive and only loosely define their priorities. Some donor countries support sector-wide approaches (SWAPs) and pool resource with other donor agencies; they often collaborate with health and finance ministries in recipient countries to generate a resource allocation plan. Very resource-constrained countries, such as Malawi, rely heavily on donor funding; they may set their own priorities and try to solicit funds accordingly but largely they "settle" for the funds and the programs that donors are offering. Countries that are less dependent on donor funding have the power to negotiate for their own priorities and can be selective and reject funding if the priorities do not match their own.

Some donor agencies or foundations may sift through scientific information and when a particular type of intervention is deemed effective they may broadcast a related program announcement. Community-based organizations and HIV/AIDS service providers seeking funds will adapt their vision, mission and programmatic efforts to accommodate these program announcements rather than risk investing in programs that are perhaps less fundable. Good publicity for an intervention, even without proven effectiveness, may entice donors to encourage its implementation. As a result, funding for a particular type of intervention comes in waves, driving several providers into that direction, irrespective of original priorities. For example, the "Abstinence, Be faithful, use a Condom" (ABC) campaign has gained a lot of popularity, esteem and funding in recent years, even though abstinence has not proven to be an effective means of HIV prevention [75].

Existing funding patterns may exert a strong influence on any new resource allocation. One common allocation mechanism consists of using the previous year's expenditures as a starting point. Funding tends to be incremental and interventions that were previously funded typically expect renewal of funds [76]. Eliminating or even decreasing funds to a program is often met with great resistance. Therefore, historical spending patterns are the greatest predictors of where resources will be allocated.

Finally, improving the allocation of HIV/AIDS resources requires an understanding of the impact of the current funding, which proves difficult to measure. For example, it is hard to discern whether a decline in disease prevalence results from a natural peak in the epidemic or from interventions and control policies. There are also multiple sources of HIV/AIDS intervention funds, and disaggregating the confounded effect of several programs is problematic since the interaction of program outcomes cannot be evaluated outside of controlled study environments. For example, HIV incidence in San Francisco has rebounded [77]. It is difficult to assess the extent to which the rebound is a result of a decrease in social marketing, prevention message fatigue, belief that ARVs reduce transmission or increase in methamphetamine use. Therefore, it becomes difficult to determine which interventions should no longer be funded and which require additional funds and renewed effort.

#### Recommendations

Thorough knowledge of potential interventions, as well as their expected impacts, duration and costs, can alleviate these funding concerns. If assumptions about these input variables must be made to enable the resource allocation model, then references and documentation are essential to avoid either resistance to change or the propensity to change too quickly. When setting existing allocations as a starting point and using models only to allocate additional funds, the health outcomes gained are relatively small and the full potential value of resource allocation models is not recognized by decision-makers. When models are used to reallocate all resources, any dramatic change to existing allocations is likely to meet resistance. However, models can be used to highlight the trade-offs between these two extreme positions and to find a middle ground that is acceptable to all stakeholders: the funding agencies, the recipient, and the service providers [20].

## Summary

AIDS policy models can provide important input to the HIV/AIDS policymaking process [78]. This paper has illustrated a range of ways in which the use of HIV/AIDS resource allocation models can be increased. Our analysis is less concerned with modelling techniques; rather, we emphasize the role of modellers in alleviating the barriers to the adoption of models and increasing the contribution of formal models to the overall resource allocation process.

Our key recommendation for improving the applicability of HIV/AIDS resource allocation models is to treat model development as a process that involves all interest groups and end users in regular meetings. The modellers provide their technical expertise but also act as educators and facilitators for all parties involved. Models require making potentially hidden assumptions explicit, and they provide powerful scenario analysis features to explore different options without risk.

Model development begins with the simplest possible model and proceeds to create a hierarchy of increasingly complex models. Each addition of complexity is suggested by sensitivity analysis and discussed by all interest groups, including feasibility and data requirement. Data requirements and accessibility are, however, not the only disadvantages of highly complex models. In many situations, simpler models provide clearer insights, allow faster sensitivity analysis and are more robust to stochastic variation.

Such a process-based approach creates a framework in which all the difficulties addressed in the previous sections can be addresses effectively and efficiently. Through continuous interaction with all groups, the modellers understand the environment in which users and decision-makers are operating. This includes knowledge of the stakeholders so that the vision on key issues and requirements, as well as potential buy-in, can be solicited from each group. Early consultation may also alleviate some concerns of those who will be asked to implement the model results by addressing issues such as social and ethical considerations, data sources and uncertainty, and stakeholder perspectives at the outset. While the requirement for the modellers to fully understand the context of a decision is not a novel recommendation, it bears repeating since it remains a demonstrable problem. Formal decision support models for HIV/AIDS resource allocation are required, but they should be contextualized and sensitive to societal concerns and decision-makers' realities. In parallel, decision-makers need to be willing to learn about the powers and limitations of models, in order to avoid abuse of models and their recommendations.

Some modellers may believe that their models should be built to reflect "proper" decision methodology and that current decision processes are flawed. Their aim is to change governmental and societal approaches to decision-making, moving it towards a more rational and/or utilitarian approach. While this vision is vital to foster growth and development, their model may prove difficult to implement in the current decision context. Therefore, modellers should assess how removed their models are from the current reality and balance their choice of an ideal vision with that of a practical tool for implementation.

Models cannot - and arguably should not - capture all of the qualitative and quantitative intricacies that surround a decision process. Models cannot be expected to dictate resource allocation; rather, their results should be used to support the process and guide discussion and policy debates. The ability to conduct "what-if" analyses using models can help decision-makers understand the extent of the trade-offs resulting from their non-quantifiable considerations. In order for decision-makers to understand and accept the trade-offs that their decisions incur, they must be comfortable with - and have confidence in - the model.

Resource allocation models provide valuable guidance to the allocation of funds. Knowledge transfer is a prime issue - how to move the expertise and insights from the academic realm into that of policymaking and resource allocation. A first step may be to convince the policymakers of the usefulness of these models by helping them solve current problems. The desire to increase the impact of HIV/AIDS funds and to improve global health outcomes should provide the necessary impetus for modellers to enhance the applicability of their models.

## Competing interests

The authors declare that they have no competing interests.

## Authors' contributions

AL conceived the paper and wrote the initial draft. AR contributed to the design of the paper and provided several drafts. FL made several revisions to improve the manuscript.
